# Toxicity Bioassay and Cytotoxic Effects of the Benthic Marine Dinoflagellate *Amphidinium operculatum*

**DOI:** 10.3390/jox11020003

**Published:** 2021-04-27

**Authors:** Ana Luisa Mejía-Camacho, Lorena María Durán-Riveroll, Allan Douglas Cembella

**Affiliations:** 1 División de Ciencias Naturales e Ingeniería, Universidad Autónoma Metropolitana-Cuajimalpa, Santa Fe CDMX 05348, Mexico; 2153031980@cua.uam.mx; 2 CONACyT-Departamento de Biotecnología Marina, Centro de Investigación Científica y de Educación Superior de Ensenada, B.C. Ensenada, Baja California 22860, Mexico; 3 Alfred-Wegener-Institut, Helmholtz-Zentrum für Polar-und Meeresforschung, 27570 Bremerhaven, Germany

**Keywords:** cytotoxicity, polyketide toxins, amphidinolide, hemolytic activity, anti-cancer

## Abstract

Benthic dinoflagellates produce a wide array of bioactive compounds, primarily polyketides, that cause toxic effects on human consumers of seafood and perhaps mediate species interactions in the benthic microenvironment. This study assesses toxic and other bioactive effects of the benthic dinoflagellate *Amphidinium operculatum* (strain AA60) in two targeted bioassays. The brine shrimp (*Artemia salina*) bioassay revealed lethal effects of direct exposure to live dinoflagellate cells (Treatment A) and even higher potency with ethanolic extracts of lysed cells (Treatment D). There were no inimical bioactive effects of components released to the aqueous growth medium (Treatment B) or from aqueous cell lysates (Treatment C). The hypothesis that released bioactive compounds provide a chemical defense against metazoan grazers is therefore not supported by these results. The cytotoxic effect of ethanolic crude extracts of this dinoflagellate exhibited mild to high growth reduction effects on six human cancer cell lines. In particular, crude cell-free extracts proved highly growth-inhibitory activity towards breast and lung cancer cell lines MCF-7 and SKLU-1, respectively. Preliminary anti-cancer results indicate that natural bioactive compounds from *Amphidinium* are worthy of structural characterization and further toxicological investigation as potential therapeutants.

## 1. Introduction

Benthic marine dinoflagellates are key primary producers and a critical component of trophic webs, particularly in tropical and sub-tropical ecosystems, such as coral reefs [1]. Species are most commonly found epibenthically, attached to macroalgae, seagrass, and detritus or as epiphytes upon hard surfaces or sediments. Benthic dinoflagellates produce a wide array of bioactive compounds, which act as toxins for human consumers of seafood when accumulated via the marine food chain and may also have toxic or allelochemical effects on other benthic organisms [2]. Benthic dinoflagellates are notorious as causative agents of seafood poisoning syndromes, most prominently ciguatera fish poisoning (CFP) and diarrheic shellfish poisoning (DSP). Known toxigenic benthic dinoflagellates are represented among more than a dozen species that produce polyketide-derived bioactive compounds [2,3,4]. These polyether compounds pose a known or potential risk to human health, and include okadaic acid and dinophysistoxins, ciguatoxins, maitotoxins, cooliatoxins, palytoxins, etc., as well as other macrolides of uncertain toxicity [5,6,7,8].

The cosmopolitan naked dinoflagellate genus *Amphidinium* Claparède et Lachmann is widely distributed in temperate, sub-tropical and tropical marine waters, occurring in either free-living or endosymbiotic states. Many *Amphidinium*, including the type species *A. operculatum* Claparède et Lachmann, are often amongst the most abundant dinoflagellates in benthic ecosystems [8]. These epibenthic species can be found attached to substrates such as macrophytes or corals or dwelling upon or in the sand. *Amphidinium operculatum* is occasionally planktonic through disturbance of its benthic habitat by storm winds or currents.

Some *Amphidinium* species, most notably *A. carterae, A. gibbosum, A. massartii* and *A. operculatum*, synthesize polyketides belonging to three structural sub-classes [9]: macrolides; short linear polyketides, and long-chain polyketides. Although toxicologically poorly characterized, these polyketides are often shown to be biologically active. Several *Amphidinium* polyketides have demonstrated cytotoxic and/or hemolytic activity against other microorganisms [10,11,12], and have even been linked to mortality in fish species [6,13,14]. More specifically, certain amphidinins [15,16], amphidinoketides [17], amphidinolides [18,19], amphidinolactone [19,20,21], caribenolide I [22], and iriomoteolides [19,23,24,25], have shown cytotoxicity against human tumor cells in vitro. Colon tumor cell line HCT and epidermoid carcinoma KB cells have been particularly responsive [26]. Accordingly, these dinoflagellate polyketides have gained attention from a biotechnological perspective due to their promising anti-cancer properties [4,12,26].

Unfortunately, much of the early work on the natural products chemistry and structural elucidation of polyketides was based upon cultured *Amphidinium* isolates of uncertain or unspecified taxonomic identification at the species level. Given the uncertainty in *Amphidinium* species assignment, and high variability and diversity among strains in capability to biosynthesize polyketides [8,12,27], it is essential to identify and characterize the strain first. Then the biological activity of the extracts should be screened, and finally the respective secondary metabolites identified and purified for further bioactivity assessment.

Primary toxicological screening for potential bioactivity effects (i.e., chemical defense) in predator-prey interactions can be conducted by exposure of target species to whole cells or extracts of the putative elicitor. Standard test subjects include the brine shrimp *Artemia salina*. This crustacean is widely used in ecotoxicological trials because of its ease of maintenance in the laboratory, short life cycle, high fecundity, and adaptability to different nutrient regimes [28,29].

Numerous published studies on in vitro assays with cancer cell lines have indicated the high capacity of secondary metabolites from dinoflagellates to inhibit cancer cell growth. A few of these novel therapeutants are in the final phases of clinical trials or in preliminary market phase, indicating high promise for dinoflagellate-derived metabolites as anticancer agents [4,30]. For decades, cancer drug discovery has been based on screening tools with cytotoxicity assays, e.g., employing colorimetric detection methods [31]. The sulforhodamine-B (SRB) assay is widely used as a rapid screening method, based on measurement of cellular protein content to estimate cellular inhibition (%) resulting from application of the test analyte [32].

Bioactive compounds produced by *Amphidinium* species have been barely explored regarding allelochemical effects in interspecies interaction in natural ecosystems, much less than even their therapeutic potential. This current study evaluated the bioactivity (“toxicity”) of a cultured strain (AA60) of *A. operculatum* isolated from the Veracruz Reef System (VRS), Gulf of Mexico, against two alternative target groups. Bioassays of *Amphidinium* with the brine shrimp *Artemia salina* were carried out to determine the general toxicity effect elicited by direct exposure to dinoflagellate cells or various extracts of cultured cells [6,8,33,34]. The brine shrimp response was evaluated to establish the general mechanism and effect of toxin release to the micro-environment, e.g., as a potential grazing inhibitor. In parallel experiments, cytotoxicity screening of extracts against six cancer cell lines was conducted to assess preliminary activity of crude fractions containing unknown bioactive components with biotechnological potential as anti-cancer therapeutants.

## 2. Materials and Methods

### 2.1. Dinoflagellate Isolation and Maintenance

Cells of *Amphidinium operculatum* were isolated from seaweed (*Padina* sp.) sampled from a buoy within the Veracruz Reef System (VRS) (Veracruz, Mexico, 19°11′54.10” N, 96° 4′0.70” W). Live seaweed samples were transported with site water in 50 mL conical plastic centrifuge tubes with ice packs to maintain ambient temperature around 24 °C during the 12 h transport to the laboratory. Substrate specimens and surrounding medium were examined for colonizing dinoflagellates in Petri plates under a stereo-dissecting microscope (Discovery.V8, Zeiss, Göttingen, Germany). Substrates were gently brushed and single-cells of epibenthic dinoflagellates were isolated by micropipette into sterile 96-well microplates containing 300 µL 50%-strength GSe growth medium [35] (modified without soil extract) prepared from autoclaved (121 °C, 15 min) seawater filtered through sand, activated carbon and 1 µm-cartridge-filters. The growth medium, supplemented with GeO_2_ (final concentration: 2.5 mg L^−1^) [36] to inhibit diatom growth, was prepared from heat-sterilized seawater stock at salinity 36. Clonal isolates were cultured at 25 ± 1 °C on a 12:12 h light:dark cycle and illumination of 50 µmol photons m^−2^ s^−1^. Well established dinoflagellate isolates were transferred to 250 mL Erlenmeyer flasks with full strength modified GSe medium and maintained as reference cultures under the conditions stated above.

Cultures for experimental biomass production and bioassay testing were started with a 15 mL inoculum in 250 mL Erlenmeyer borosilicate flasks. Cell density was monitored until maximal optical density by visible inspection. Final cell counts were performed for harvest and extraction in late exponential growth to stationary phase transition (*ca*. two weeks to 30 d). Cell density at harvest was assessed by taking a 2 mL culture sample diluted 1:10 with GSe, then fixed with acidic Lugol’s iodine solution, and counted in a Sedgewick-Rafter counting chamber.

### 2.2. Dinoflagellate Identification

Clonal isolate AA60 in the present study was assigned to *A. operculatum* Claparède & Lachmann by detailed photonic and scanning electron microscopy (SEM). Briefly, 50 µL of live cell culture was mixed with 50 µL of seawater and visualized on a light microscope (Axio Observer A1, Zeiss, Oberkochen, Germany) at 200–1000ᵡ magnification. Photomicrographic images were recorded after visual inspection of cells. SEM images were obtained by following the method of Pérez-López et al. [37] for the genus *Amphidinium*. Cells were observed on a JSM 6360-LV scanning electron microscope (JEOL Tokyo, Japan).

The *Amphidinium* species identity was confirmed by sequencing the large subunit (LSU) and ITS regions of the rDNA gene. DNA was extracted by a modified CTAB method [9,14,38,39] and purified with DNA Clean & Concentrator (Zymo Research, Irvine, CA, USA) following the manufacturer’s recommendations. The polymerase chain reaction (PCR) was performed with the primers D1R [40] and 28-1483 [41] using the Phire Plant Direct PCR Kit (Thermo Fisher Scientific, Waltham, MA, USA) according to the manufacturer’s instructions. PCR thermocycler conditions were set as follows: in a PCR machine Flexigene (Techne, Staffordshire, UK), 98 °C for 5 min, 98 °C for 20 s, followed by 40 cycles at 48 °C for 30 s, and finally 72 °C for 1 min and 20 s. Sequencing reactions were analyzed with the Genetic Analyzer ABI Prism 3100 (Applied Biosystems, Foster, CA, USA).

### 2.3. Preparation of Ethanolic Extracts of Cultured Amphidinium Cells

*A. operculatum* cell culture (13 mL) was harvested in late exponential to early stationary growth phase by centrifugation at 35× *g* for 10 min (Solbat J12, Puebla, Mexico) to yield a loose cell concentrate with minimal cell damage. The supernatant was removed, and the cell concentrate was stored at −65 °C. For freeze-thaw extraction, the cell concentrate was thawed and then centrifuged at 1500×* g* for 5 min at room temperature (24 °C). The cell pellet was washed with 1 mL of cold seawater (4 °C), centrifuged again at 1500× *g* for 5 min and stored for 24 h at −65 °C. The pellet was extracted with 2 mL absolute EtOH (ACS grade, Merck Millipore, Darmstadt, Germany) and frozen for 30 min at −65 °C. Afterwards, the cell suspension was vortex-mixed for 30 s. The freeze and thaw cycles were repeated until complete cell disruption was verified by observations with a stereo-microscope (Discovery V8, Zeiss). Next, the sample was centrifuged at 6720×* g* for 5 min and the supernatant was filtered with a 0.2 µm syringe filter (Puradisc, Whatman, Maidstone, UK) into a 2 mL microtube. Finally, the crude ethanolic extract was lyophilized (LabConco Freeze Dry, Kansas City, KS, USA) to yield an EtOH-free extract and stored at −65 °C until use as Treatment D in the acute toxicity bioassay and the SRB assay.

### 2.4. Acute Toxicity Artemia Assay

*Artemia salina* dry cysts (Eclosionazul, Mexico, Mexico) were incubated in 2.5% NaCl medium under continuous illumination and aeration at room temperature. After 48 h the hatched nauplii were transferred to a Petri dish with fresh saline medium to facilitate collection.

Acute toxicity tests were performed with four alternative fractions applied to live brine shrimp nauplii: Treatment A with live *Amphidinium* cells; Treatment B with extracellular growth medium (minus *Amphidinium* cells); Treatment C with crude *Amphidinium* cell lysate; and Treatment D with ethanolic extract of cultured *Amphidinium* cells.

Treatment A was designed to assess the effects of direct exposure of brine shrimp nauplii to intact *Amphidinium* cells. *Amphidinium* cells (1 mL transfer) were grown and harvested from 125 mL of modified GSe medium under the conditions specified in Section 2.1. A geometric-model dilution series [42] was prepared in modified GSe medium to yield the following concentrations: 0.3, 1.0, 3.2, 10.0, 31.6, and 100.0 × 10^3^ cells mL^−1^.

In Treatment B, brine shrimp nauplii were exposed to the growth medium without *Amphidinium* cells to examine the effects of extracellular metabolites leaked or excreted from apparently healthy cells. The supernatant of *Amphidinium* culture samples prepared as for Treatment A was retained after gentle centrifugation (*ca*. 3000× *g*) to yield the leaked or excreted metabolites from cell equivalents corresponding to the dilution series for Treatment A.

In Treatment C, aqueous lysates of cultured *Amphidinium* cells were prepared to test intracellular metabolite effects on brine shrimp nauplii. The pellets obtained from the centrifuged Treatment B samples were resuspended in 2 mL modified GSe medium and homogenized (Polytron homogenizer, Thomas Scientific, Swedesboro, NJ, USA) for 30 s on ice. To assure cell disruption, samples were vortex-mixed for approximately 40 s and treated under 3 freeze-thaw cycles, followed by ultrasonication (USR-1, Julabo, Allentown, PA, USA) on ice. Finally, the pooled extracts were homogenized and pipetted to yield a geometric dilution series corresponding to cell equivalents for Treatment A.

In Treatment D, freeze-dried ethanolic extract of cultured *Amphidinium* cells was applied (EtOH-free) to brine shrimp to assay the effects of EtOH-soluble intracellular components on behavior and mortality of nauplii. The lyophilized ethanolic extract was dissolved in 1 mL modified GSe seawater medium by ultrasonication to yield a final concentration of 4.3 mg mL^−1^ equivalent to 8.9 × 10^5^ cells mL^−1^. For the experimental assay, the stock extract was diluted to the following: 153.0, 114.8, 86.1, 64.6, 48.4, 36.3, 27.2, 20.4, and 15.3 μg mL^−1^, based on a geometric-model dilution series [42], equivalent to 31.6, 23.7, 17.8, 13.3, 10.0, 7.5, 5.6, 4.2, and 3.2 × 10^3^ cells mL^−1^, respectively.

Bioassays were conducted in triplicate for each treatment in 96-well microplates containing 100 μL of 2.5% NaCl medium with 10 nauplii and addition of 100 μL of each treatment. GSe seawater medium and 2.5% NaCl solution served as negative controls (*n* = 3). The microplates were incubated under normal laboratory conditions (12:12 h artificial daylight:dark, 24 °C) for 24 h. After this time, dead nauplii were counted, and then 100 μL of 1:1 EtOH:acetone were added to each well to sacrifice the surviving nauplii to determine the total number, in case there were more than 10 nauplii in the test wells.

### 2.5. Dose-Response Curve

A dose-response curve was plotted to assess LC_50_ mortality, the treatment concentration yielding death of 50% of the test subjects under the specified experimental conditions. The percentage of nauplii mortality was calculated as follows:

% Mortality = (Nm/Nt) × 100(1)

where Nm represents the number of dead nauplii and Nt the total nauplii [43]. Mean mortality percentage for each concentration tested was adjusted to a five-parameter logistic equation with GraphPad Prism version 8.3 (GraphPad Software, San Diego, CA, USA).

### 2.6. Cell Line Culture and Assays

Six human cancer cell lines were assayed for effects on growth after exposure to ethanolic extracts of *Amphidinium* cultures: U251 (human glioblastoma), PC-3 (human prostatic adenocarcinoma), K562 (human erythroleukemia), HCT-15 (human colorectal adenocarcinoma), MCF-7 (human mammary adenocarcinoma), SKLU-1 (human lung adenocarcinoma). All cancer cell lines were obtained from the National Cancer Institute (NCI, Bethesda, MD, USA). The cell lines were cultured in RPMI-1640 medium [44] supplemented with 10% fetal bovine serum, 2 mM L-glutamine, 10,000 units mL^−1^ penicillin G sodium, 10,000 μg mL^−1^ streptomycin sulfate and 25 μg mL^−1^ Amphotericin B (Gibco, Waltham, MA, USA), and 1% non-essential amino acids (Gibco) [44]. The cell lines were maintained at 37 °C in humidified atmosphere with 5% CO_2_. The viability of the cells in the experiments exceed 95% as determined with Trypan blue [44].

### 2.7. Sulforhodamine B Assay

Cytotoxicity of the ethanolic extract of *A. operculatum* cells was evaluated with a colorimetric screening method based on the detection of cellular protein with sulforhodamine B (SRB). This microculture assay was configured to measure cell growth, as described in the protocols established by NCI [44]. The tissue culture cells were removed from the culture flasks by treatment with trypsin and diluted with fresh medium. A fixed volume (100 μL) of cell suspension, containing 5 to 10 × 10^3^ cells per well, was pipetted into 96-well sterile microtiter plates, and then incubated at 37 °C for 24 h in a 5% CO_2_ atmosphere. The lyophilized ethanolic extract (prepared as described in Section 2.3) was diluted with PBS buffer. Subsequently, 100 μL of the diluted ethanol extract were added to each well.

The cultured cells were exposed to the extract for 48 h at a final concentration of 24.7 μg mL^−1^, equivalent to 5.1 × 10^3^ cells mL^−1^. PBS buffer was used as negative control. After incubation, cells were fixed to the plastic substratum by addition of 50 μL of cold 50% aqueous trichloroacetic acid. The plates were incubated at 4 °C for 1 h, washed with distilled water, and air-dried. The trichloroacetic-acid-fixed cells were stained by addition of 0.4% SRB. Free SRB solution was then removed by washing with 1% aqueous acetic acid. The plates were air-dried, and the bound dye was solubilized by addition of 10 mM unbuffered Tris base (100 μL). The plates were placed on a shaker for 10 min, and the absorption at 515 nm was determined with an ELISA plate reader (Bio-Tex Instruments, TX, USA). Percentage of inhibition was calculated as described by Vichai and collaborators [32]. The results were graphed with GraphPad Prism version 8.3.

### 2.8. Statistics

To compare the differences between the dose-response curves obtained from the acute toxicity *Artemia* assay (Section 2.4), the confidence interval (α = 0.05) was determined with GraphPad Prism version 8.3. One-way ANOVA (α = 0.05) was used to assess the inhibitory effect of the ethanolic extract on the cancer cell lines tested. An a posteriori Tukey test (α = 0.05) was applied to establish the significant differences between effects on cancer-cell lines. The ANOVA and Tukey’s test were performed with Microsoft Excel version 15.13.3. (Microsoft Corporation, Redmond, WA, USA)

## 3. Results and Discussion

### 3.1. Dinoflagellate Description and Identification

**Figure 1:**
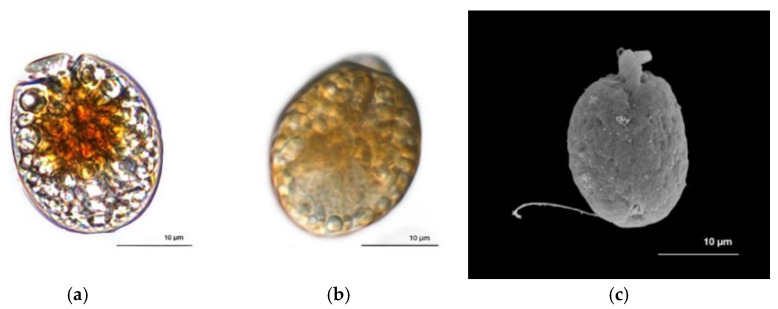
*Amphidinium operculatum* AA60. (**a**) and (**b**) Dorsal view by photonic microscopy. (**c**) Ventral view by scanning electron microscopy. Scale bar = 10 μm.

*A. operculatum* (AA60) cells (*n* = 6) are ellipsoidal in shape (mean length: 27.48 ± 4.59 µm; mean width: 18.26 ± 2.73 µm) and correspond to the classic description of the species [45]. The reduced epicone is located at the center apex of the cell, but oriented toward the left side (Figure 1a). Multiple orange-yellow chloroplasts are found near the center of the cell, radiating to the periphery (Figure 1a). The nucleus is located in the posterior part of the cell (Figure 1b). Posterior trailing flagellar insertion is near the epicone on the central axis of the cell (Figure 1c).

The LSU and ITS rDNA sequences align well with those provided in the literature [38], and leave no doubt regarding the species identity. The sequences of the LSU and ITS regions of the rDNA gene of AA60 are available in the DNA repository database (GenBank accession number MT325891).

### 3.2. Acute Toxicity Assay

*A. operculatum* was toxic towards *Artemia salina* nauplii when they were exposed to whole cell culture (Treatment A) and ethanolic extract (Treatment D). The effect on the nauplii was almost immediate after applying the most concentrated dilutions of ethanolic extract (at 153, 115, and 86 μg mL^−1^, equivalent to 31.6, 23.7, and 17.8 × 10^3^ cells mL^−1^, respectively). The toxic metabolites of *A. operculatum* act rapidly, but the response is dosage-threshold dependent upon the cell equivalents. In contrast, when nauplii were treated with cell-free extracellular medium (Treatment B) and cell lysate (Treatment C) of *A. operculatum* AA60, no nauplii mortality was observed (as in the negative controls) throughout the dilution series.

**Figure 2:**
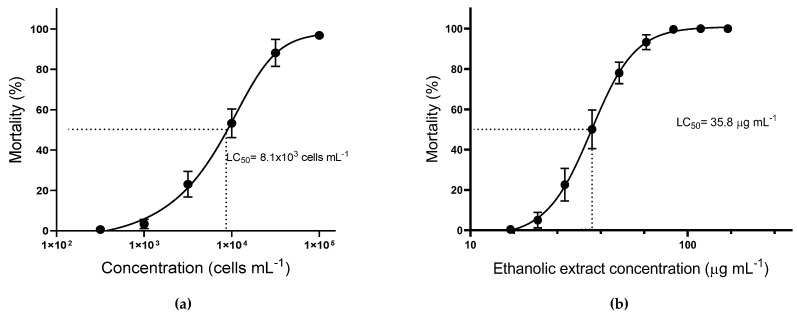
Mortality of *Artemia salina* nauplii after 24 h of incubation (mean ± sem; *n* = 3). (**a**) Treatment A, *Artemia* exposed to different *A. operculatum* cell densities; (**b**) Treatment D; *Artemia* exposed to different *A. operculatum* ethanolic extract dosages.

Since nauplii mortality was only observed with Treatments A and D, the lethal concentration for 50% of the nauplii (LC_50_) was assessed only for these treatments (Figure 2). The toxicity of intact live cells of *A. operculatum* against brine shrimp was significantly lower (α = 0.05) (LC_50_ = 8.1 ± 0.8 × 10^3^ cells mL^−1^) than with the ethanolic extract (LC_50_ = 35.8 ± 0.8 μg mL^−1^, equivalent to 7.4 ± 0.2 × 10^3^ cells mL^−1^). Nevertheless, no significant differences were found between the dose-response curves for Treatments A and D.

### 3.3. Ecotoxicological Assessment of A. operculatum

The biosynthetic pathways and extracellular release mechanisms for bioactive compounds produced by *Amphidinium* species are poorly understood. There is even less knowledge regarding their role in species interactions in the environment. In the current bioassay experiments, the brine shrimp *Artemia salina* was shown to be negatively affected by direct exposure to live cells of *A. operculatum* AA60 and to ethanolic extract but insensitive to application of cell lysate and cell-free growth medium. Since living *Amphidinium* cells were toxic, the brine shrimp are susceptible to toxic intracellular compounds released only upon digestive release from dinoflagellate cells after grazing, but insensitive to the inactivated or matrix-bound components in cell lysates.

Alternatively, the lethal effect of exposure to actively metabolizing dinoflagellate cells but not cell lysates may be due to the production of extracellular mucus [46]. Exopolysaccharide (“mucus”) is produced by a wide variety of microalgae and cyanobacteria, particularly in benthic environments. Such mucus may play multiple roles in cell attachment to substrates, inhibition of bacterial colonization and resistance to predators [47]. For example, the benthic toxigenic dinoflagellate *Ostreopsis ovata* can cause sea urchin (*Paracentrotus lividus*) larval mortality at high cell densities by completely covering the larvae, thereby creating a mechanical barrier affecting larval swimming [48].

Mucus production by benthic *Amphidinium* species is well known [47]. Mortality of crustacean nauplii in the presence of whole live cells in the bioassays with *A. operculatum* could be due to purely hydromechanical effects. Mucus sheathes the nauplii and disrupts its swimming appendages and/or inhibits oxygen transfer (“smothering”) [49].

Clumping of cells by mucus aggregates may cause impaired feeding by obstructing the gastrointestinal tract of *Artemia*. Moreira-González and collaborators [46,50] inferred this based on their observations on the effect of different species of *Amphidinium* on *Artemia salina*. No detailed investigation of grazing rate or behavior was conducted over the acute toxicity trials in the experiments presented herein. Yet we did confirm by microscopic observations that the *A. operculatum* AA60 produced abundant mucus and often clumps of cells. Given the size of *Artemia* nauplii (400 to 500 μm [51]) at the stage at which the current experiments were carried out, they should have been able to filter food particles varying from 1 to 50 μm [52]. This is within the size range of *A. operculatum* AA60 cells, but mucus aggregates producing clumps of cells could make it more difficult for predators to ingest them [53]. In any case, there is circumstantial evidence that metabolic impairment and mortalities of crustaceans by exposure to naked dinoflagellates is mediated by a synergistic chemical mechanism rather than exclusively as a hydromechanical response to mucus production. Mucus can provide enhanced exposure to noxious or toxic bioactive compounds retained within the hydrogel. This magnifies the surface contact area, intensifying and prolonging the exposure [54]. In the case of *A. operculatum*, a synergistic toxic effect on the membrane ATPase pump may account for the rapid effect of benthic dinoflagellate cells that come in contact with larvae [19]. This rapid response on *Artemia* nauplii is consistent with the fact that the molecular target of some amphidinolides produced by *Amphidinium* involves functional ATPase [19].

Exposure to cells of the mucus-producing naked dinoflagellate formerly called *Gyrodinium corsicum* affects the motor functions of the copepod *Acartia grani* thereby causing paralysis [55]. *Gyrodinium corsicum* is now recognized as a synonym of *Karlodinium corsicum* (Paulmier, Berland, Billard & Nezan) Siano & Zingone [56]. This species is a known producer of potent polyether karlotoxins associated with fish mortalities via membrane disruption [57]. The dinoflagellate attaches to the surface of the copepod and disrupts the mechanical and chemical sensory system of its antennae, which can enhance the direct absorption of toxic substances produced [55].

With *A. operculatum*, given that Treatment B did not affect the nauplii, strain AA60 does not release substantial amounts of targeted bioactive compounds to the surrounding medium from healthy cells. Moreover, the outcome of Treatment D on the nauplii supports the idea that *A. operculatum* keeps its lipophilic bioactive compounds intracellularly. The rapid effect of the ethanolic extract on nauplii concurs with the fast-acting mechanisms proposed for hemolytic toxic compounds from dinoflagellates [46]. The evaluation of Treatment C with cell lysate at first appears to contradict this interpretation, considering that no dead nauplii were observed. These findings could mean, however, that some potentially bioactive compounds are not water-soluble. Such compounds may be inactivated or chemically bound by the lysate matrix, thus generating no effect on the nauplii. This would explain why, in the absence of live dinoflagellate cells, nauplii mortality occurred only when the bioactive compounds were first extracted into EtOH, a better solvent than water for the less polar polyketides produced by *Amphidinium*.

Complementary research on an *A. operculatum* clone from Brazil (strain Ao-Ecpb-1) [46] has established that the toxicity effect on *Artemia*, with comparable bioassay methods, is not unique to strain AA60 from the Veracruz Reef System (VRS), Gulf of Mexico. The *A*. *operculatum* strain Ao-Ecpb-1 exhibited higher toxicity when nauplii were exposed to whole live cells than to the EtOH-soluble cell fraction [46]. In comparison with *Amphidinium* strains of *A. massartii* (LC_50_ = 31 cells mL^−1^) and *A. operculatum* (LC_50_ = 347 cells mL^−1^) isolated from Cuba and Brazil, respectively [46], the LC_50_ of strain AA60 (8065 cells mL^−1^) was much higher. Hence AA60 is less toxic on a per cell basis. The much lower cell toxicity for the *A. operculatum* populations from the VRS may pose a relatively lower ecotoxicological risk to human health. Yet it is premature to conclude such an assessment without screening multiple isolates from the region for strain-specific variability.

### 3.4. SRB Assay on Cell Lines

**Figure 3:**
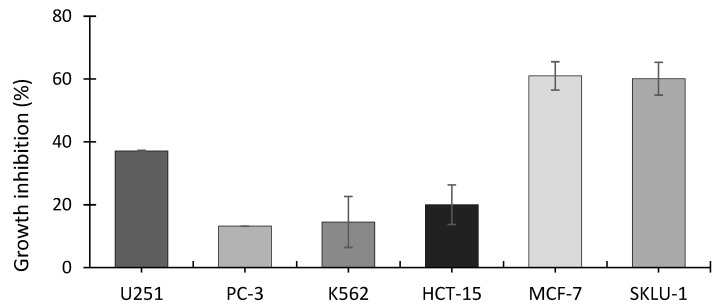
Growth inhibition of cell lines exposed to *A. operculatum* ethanolic extract at 24.7 μg mL^−1^ (equivalent to 5110 cells mL^−1^). Histograms show the mean ± sem (*n* = 3) of inhibition (%). Cell type: U251 (human glioblastoma); PC-3 (human prostatic adenocarcinoma); K562 (human erythroleukemia); HCT-15 (human colorectal adenocarcinoma); MCF-7 (human mammary adenocarcinoma); SKLU-1 (human lung adenocarcinoma).

The SRB assay showed that the ethanolic extract from *A. operculatum,* at a dosage of 24.7 μg mL^−1^ (equivalent to 5110 cells mL^−1^), inhibited the growth of all the cancer cell lines tested (Figure 3). Cell lines MCF-7 (human mammary adenocarcinoma) and SKLU-1 (human lung adenocarcinoma) showed the highest inhibitory activity with around 60% cell inhibition, followed by U251 (human glioblastoma) (37.1%), HCT-15 (human colorectal adenocarcinoma) (20%), K562 (human erythroleukemia) (14.5%), and PC-3 (human prostatic adenocarcinoma) (13.2%). Significantly higher inhibition was found between MCF-7 and SKLU-1 and all the other cell lines (Tukey’s post-hoc test); *p*-value < 0.00001 for MCF-7 and *p*-value ≤ 0.00001 SKLU-1, compared with PC-3, K562, and HCT-15.MCF-7 (*p*-value < 0.00092) and SKLU-1 (*p*-value = 0.00128) were also both significantly more growth inhibited than U251. No significant differences in inhibition were found among PC-3, K562 and HCT-15 cell lines.

### 3.5. Cytotoxicity of A. operculatum on Cancer Cell Lines

Extracts of *Amphidinium* cells have received recent attention due to cytotoxic properties, especially from strains of *A. carterae*, *A. massartii* and *A. operculatum*, against selected target planktonic organisms and cell lines. Several bioactive compounds produced by *Amphidinium* have been isolated and tested (albeit not exhaustively screened) against cancer cells with favorable results, as in the case of amphidinolides [18,19], caribenolides [22] and amphidinols [4,9,26]. In this context, we anticipated that the ethanolic extract from *A. operculatum* AA60 may also inhibit cancer cell growth; this proved to be the case at an assay dose of 24.7 μg mL^−1^. Prior to this study, there were no reports on evaluation of crude ethanolic extracts of *Amphidinium* cells on cancer cell lines, but there were a few investigations on effects of methanolic cell extracts. Cytotoxicity of methanolic crude extract from two *A. operculatum* strains collected from Jeju Island, Korea has been evaluated with promielitic cell line HL-60 of human leukemia. At 50 μg mL^−1^, the extract of both strains caused strong growth inhibition between 40 and 60% in HL-60 cell line, whereas at 25 μg mL^−1^ growth inhibition was ca. 30% [4,11]. On this basis, the methanolic extracts of the strains isolated from Korea were apparently more cytotoxic than the ethanolic extract of the *A. operculatum* AA60 on a leukemia cell line. Note the moderate inhibition of growth of K562 (only 14.5%) in response to AA60. The slight polarity differences between methanolic versus ethanolic exaction is not expected to yield dissimilar fractions of putatively bioactive polyether compounds. Nevertheless, this dosage comparison must be interpreted cautiously because of the undefined differences in the leukemia cell lines.

The growth inhibition effect of extract of *A*. *operculatum* AA60 was particularly pronounced on cell lines MCF-7 (human mammary adenocarcinoma) and SKLU-1 (human lung adenocarcinoma), both with about 60% inhibition. There are no previous reports in which a crude extract of *A. operculatum* was tested against MCF-7. However, a methanolic extract of *A. carterae* cells has been assayed against MCF-7; at high dosage (175 µg mL^−1^), it proved to be remarkably potent, with 100% cell growth inhibition [58]. The high extract dose tested was almost seven times higher than the concentration in the current study, therefore the dose-response effects on growth are not directly comparable.

Methanolic extract of *A. carterae* cells inhibited growth of human lung adenocarcinoma cells (A549) by approximately 40% at a dosage of 50 μg mL^−1^ [59]. Nevertheless, the result obtained herein with the ethanolic extract against SKLU-1 (lung cancer) reveals that the ethanolic extract from *A. operculatum* strain AA60 is more cytotoxic against a similar lung cancer cell line. The extract from AA60 inhibited 20% more at half the dosage in the study by Samarakoon et al. [59] with *A. carterae* cells.

Direct comparisons of potency must be interpreted with extreme caution even when homologous assay methodologies and identical target cell lines are selected. The effects of slight differences in application of assay protocols are difficult to evaluate. Moreover, direct comparison and interpretation of cell-equivalent dose-responses should be viewed critically because of differences in cell content and composition of the extracted bioactive metabolites between *Amphidinium* strains and species. The effect on cell growth inhibition by the ethanolic extract as tested could be more potent than indicated in the results presented due to the limited solubility of the bioactive compounds in aqueous PBS. As a preliminary step, screening of such crude extracts for bioactivity is vital to the determination of inhibition mechanisms. These results with *Amphidinium* extracts open the door to further investigation on the chemical composition of this crude ethanolic extract and discovery of potential therapeutants against cancer cells.

## 4. Conclusions

Healthy live cells of *Amphidinium operculatum* AA60 do not release substantial amounts of toxic compounds to the immediate microenvironment, at least at dosages lethal to potential grazing crustaceans and at environmentally realistic cell densities found in nature. The brine-shrimp assay does not support the hypothesis that these are acting as allelochemicals against metazoan predators. Despite the apparent low cell toxicity of *A. operculatum* AA60 other strains from the VRS may be more toxic. Future increase in the abundance of these dinoflagellates due to environmental regime shifts affecting coral reefs would further enhance the toxic effect on various marine invertebrates and larvae of species of ecological and economic importance. The risk to human health of known polyether compounds produced by *Amphidinium* when accumulated in seafood remains to be evaluated.

High cell densities may partially compensate for low cell potency, and total release of xenobiotics upon senescence of benthic micro-blooms may yield unrecognized but profound effects upon reef communities. Such compounds are expected to be released by mechanical cell disruption, e.g., resulting from storms or other turbulent events. These athecate dinoflagellates are rather fragile. During senescence phase of the growth cycle, cells lose membrane integrity. The exact mechanism by which these compounds are leaked or excreted into aqueous medium from healthy cells remains to be determined.

*Amphidinium* AA60 synthesizes bioactive compounds capable of inhibiting the growth of some cancer cell lines, particularly MCF-7 (human mammary adenocarcinoma) and SKLU (human lung adenocarcinoma). These preliminary results confirm the potency of mostly unknown bioactive compounds produced by the dinoflagellate *A. operculatum* and close phylogenetic relatives. For this reason, the biotechnological potential of *Amphidinium* strains should be further explored to evaluate not only the effects of crude extract, but also to screening for effects of purified fractions of known composition of bioactive molecules.

## Data Availability

The sequences of the LSU and ITS regions of the rDNA gene of AA60 are available in the DNA repository database (GenBank accession number MT325891).
